# An investigation of age dependency in Dutch and Chinese values for EQ-5D-Y

**DOI:** 10.3389/fpsyg.2023.1175402

**Published:** 2023-10-03

**Authors:** Brigitte Essers, Pei Wang, Elly Stolk, Marcel F. Jonker, Silvia Evers, Manuela Joore, Carmen Dirksen

**Affiliations:** ^1^Department of Clinical Epidemiology and Medical Technology Assessment, Maastricht University Medical Centre, Maastricht, Netherlands; ^2^Care and Public Health Research Institute (CAPHRI), Maastricht, Netherlands; ^3^School of Public Health, Fudan University, Shanghai, China; ^4^EuroQol Research Foundation, Rotterdam, Netherlands; ^5^Erasmus Choice Modelling Centre, Erasmus University Rotterdam, Rotterdam, Netherlands

**Keywords:** health state values, age-dependency, health related quality of life, children, EQ-5D-Y

## Abstract

**Aims:**

The primary aim was to explore the age dependency of health state values derived via trade-offs between health-related quality of life (HRQoL) and life years in a discrete choice experiment (DCE). The secondary aim was to explore if people weigh life years and HRQoL differently for children, adolescents, adults, and older adults.

**Methods:**

Participants from the general population of the Netherlands and China first completed a series of choice tasks offering choices between two EQ-5D-Y states with a given lifespan. The choice model captured the value of a year in full health, disutility determined by EQ-5D-Y, and a discount rate. Next, they received a slightly different choice task, offering choices between two lives that differed in HRQoL and life expectancy but produced the same number of quality-adjusted life years (QALYs). Participants were randomly assigned to fill out the survey for three or four age frames: a hypothetical person of 10, 15, 40, and 70 years (the last one only applicable to China) to allow the age dependency of the responses to be explored.

**Results:**

A total of 1,234 Dutch and 1,818 Chinese people administered the survey. Controlling for time preferences, we found that the agreement of health state values for different age frames was generally stronger in the Netherlands than in China. We found no clear pattern of differences in the QALY composition in both samples. The probability distribution over response options varied most when levels for lifespan or severity were at the extremes of the spectrum.

**Conclusion/discussion:**

The magnitude and direction of age effects on values seemed dimension- and country specific. In the Netherlands, we found a few differences in dimension-specific weights elicited for 10- and 15-year-olds compared to 40-year-olds, but the overall age dependency of values was limited. A stronger age dependency of values was observed in China, where values for 70-year-olds differed strongly from the values for other ages. The appropriateness of using existing values beyond the age range for which they were measured needs to be evaluated in the local context.

## Introduction

In recent years, the demand for pediatric multi-attribute utility instruments has grown (Chen and Ratcliffe, 2015). One of these utility instruments is the EQ-5D-Youth (EQ-5D-Y), a child-friendly version of the well-known adult questionnaire EQ-5D-3L (Wille et al., 2010). It contains the same five health dimensions, although the wording of three of them (i.e., self-care, anxiety, and usual activities) has been modified in order to fit the needs of the younger respondent. A VAS scale is also included, with endpoints of 0 (the worst health you can imagine) and 100 (the best health you can imagine). The EQ-5D-Y questionnaire can be filled out by children from the age of 8, while for children aged 4–7, a proxy version can be applied. EQ-5D-Y value sets are currently available for nine countries (Devlin et al., 2022).

Key challenges in the area of child health valuation are the impact of different perspectives, i.e., adult, adolescent, or child preferences, and the impact of different health state valuation methods (Rowen et al., 2020). The EQ-5D-Y valuation protocol requires that the general population should be asked to value the EQ-5D-Y health states as proxies for children. People no longer value the health state of a person like themselves but of a 10-year-old hypothetical child. To date, it is unknown whether the obtained values will be sensitive to the specified age of the hypothetical child (e.g., a child aged 10 or an adolescent aged 15), and if so, what framing of age is optimal.

The available evidence about the age dependency of health-related quality of life (HRQoL) values is limited. Kind et al. showed that by using the visual analog scale (VAS), the obtained values were lower for children when respondents were asked to imagine that a health state concerned a 10-year-old child compared to when they valued that state for themselves or another adult (Kind et al., 2015). These results suggest that health problems will affect a child's HRQoL more than an adult's HRQoL. However, the Kind's VAS values were obtained on a scale with the best and worst imaginable health states as the top and bottom anchors and not on the full health-dead scale required for the computation of quality-adjusted life years. Kreimeier et al. (2018) reported that TTO values for children exceed those for adults in the same health state. Shah et al. (2020) found the same result across a range of methods that all produced values on the full health-dead scale.

To better understand the age dependency of HRQoL values, we need to carefully examine the context and meaning of responses given to questions, especially when they involve a time trade-off. Because TTO values are derived from a trade-off between HRQoL and time, HRQoL values are confounded with preferences for time. As a result, differences in TTO values for adults and children have a clear interpretation: Are changes in health affecting children's HRQoL less, or are variations in time preferences impacting the difference as well? This issue needs to be investigated further in order to better understand differences in health state values for children, adolescents, adults, and/or older adults and to advance valuation methods.

The main objective of our research was to examine how age impacts the valuation of EQ-5D-Y health states using a discrete choice experiment (DCE) that included a duration attribute. The second objective was to study if there are cultural differences when valuing health states for children, adolescents, or adults. The third objective was to explore if people attach different relative weights to life years and quality of life for children, adolescents, adults, and older adults.

## Methods

### Strategy

Respondents in the Netherlands were randomized over three arms that only differed by the framing of the valuation task with respect to the age of the hypothetical person that would experience the health states: 10 years (arm 1), 15 years (arm 2), or 40 years (arm 3), representing a child, an adolescent, and an adult. The study in China adopted the same study design as used in the Netherlands and extended it with a fourth study arm focused on older adults over 70 years. This was done to increase the contrast between arms and increase knowledge of the validity and valuation of the EQ-5D in the elderly population. Respondents in both countries completed two tasks. First, they received a series of questions from a discrete choice experiment featuring EQ-5D-Y health states with an associated duration. Next, respondents received a series of questions asking about their preferences for a “QALY composition”. Details of both tasks are provided below. Approval for this study was given by the Ethics Committees of the University of Maastricht and the Institutional Review Board of Fudan University School of Public Health before the start of the study. Data collection took place between August and December 2017 in the Netherlands and between May and July 2019 in China.

### EQ-5D-Y

EQ-5D-Y is a five-dimensional measure of health-related quality of life, derived from EQ-5D (Wille et al., 2010). The included dimensions are mobility, looking after myself, doing usual activities, having pain or discomfort, and feeling worried, sad, or unhappy. Each dimension has three levels: no problems, some problems, and a lot of problems.

### Sample

In the Netherlands, respondents were recruited from a commercial panel “Panelinzicht”. Each respondent received an invitation with a link to participate in the survey. To make sure the sample was representative of the Dutch population, stratified sampling was applied. This means that three strata were defined beforehand: age (with 18 years as a minimum age), gender, and education. Based on the classification as used by Statistics Netherlands (Centraal Bureau voor de Statistiek), the eight levels of education were divided into lower, middle, and higher education. In China, the respondents were enrolled by Survey Engine, and quota sampling was used to generate a representative sample of the general adult population in terms of age and gender.

### The survey

**Figure 1:**
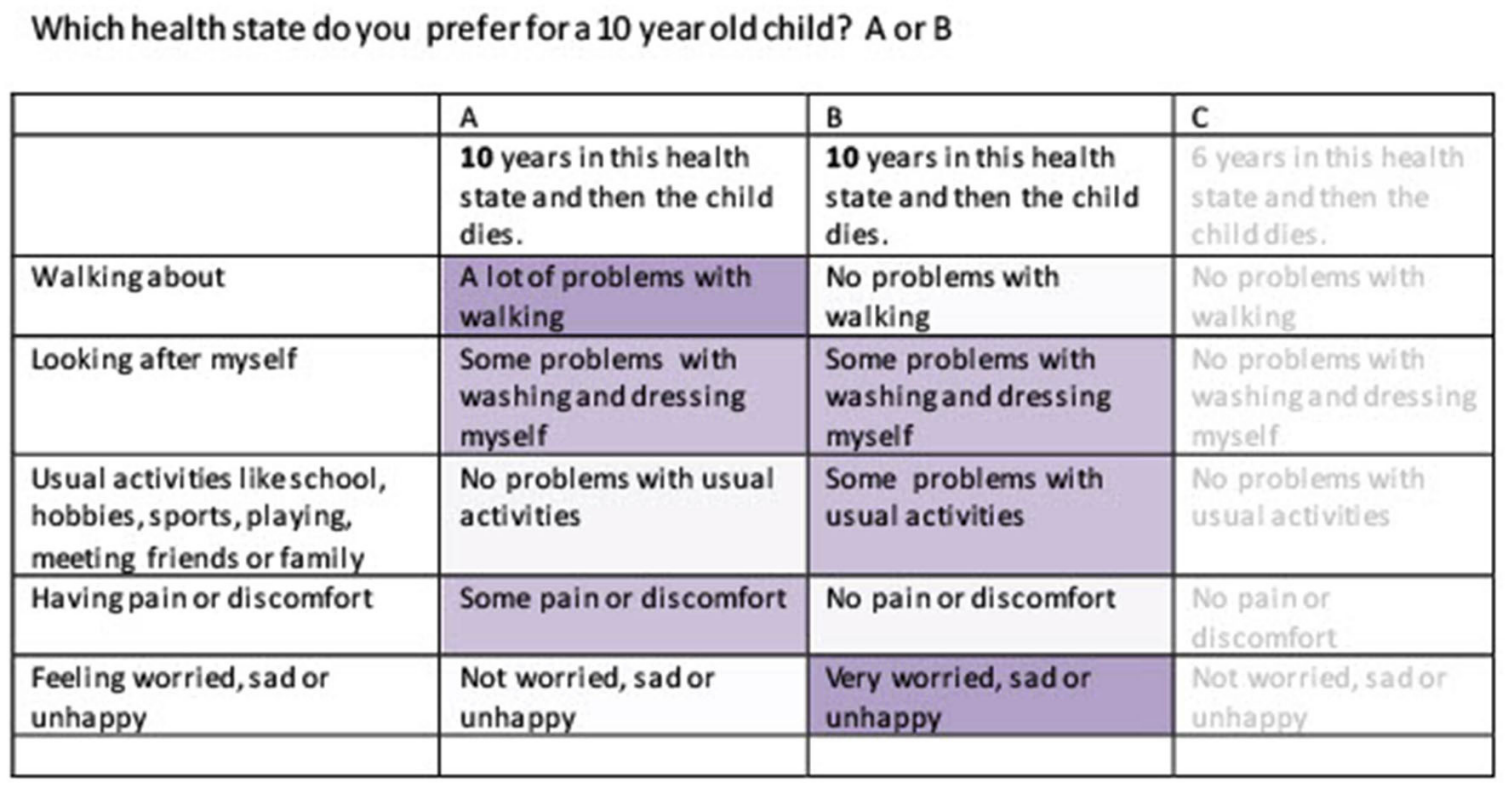
Example choice set health state A and B.

**Figure 2:**
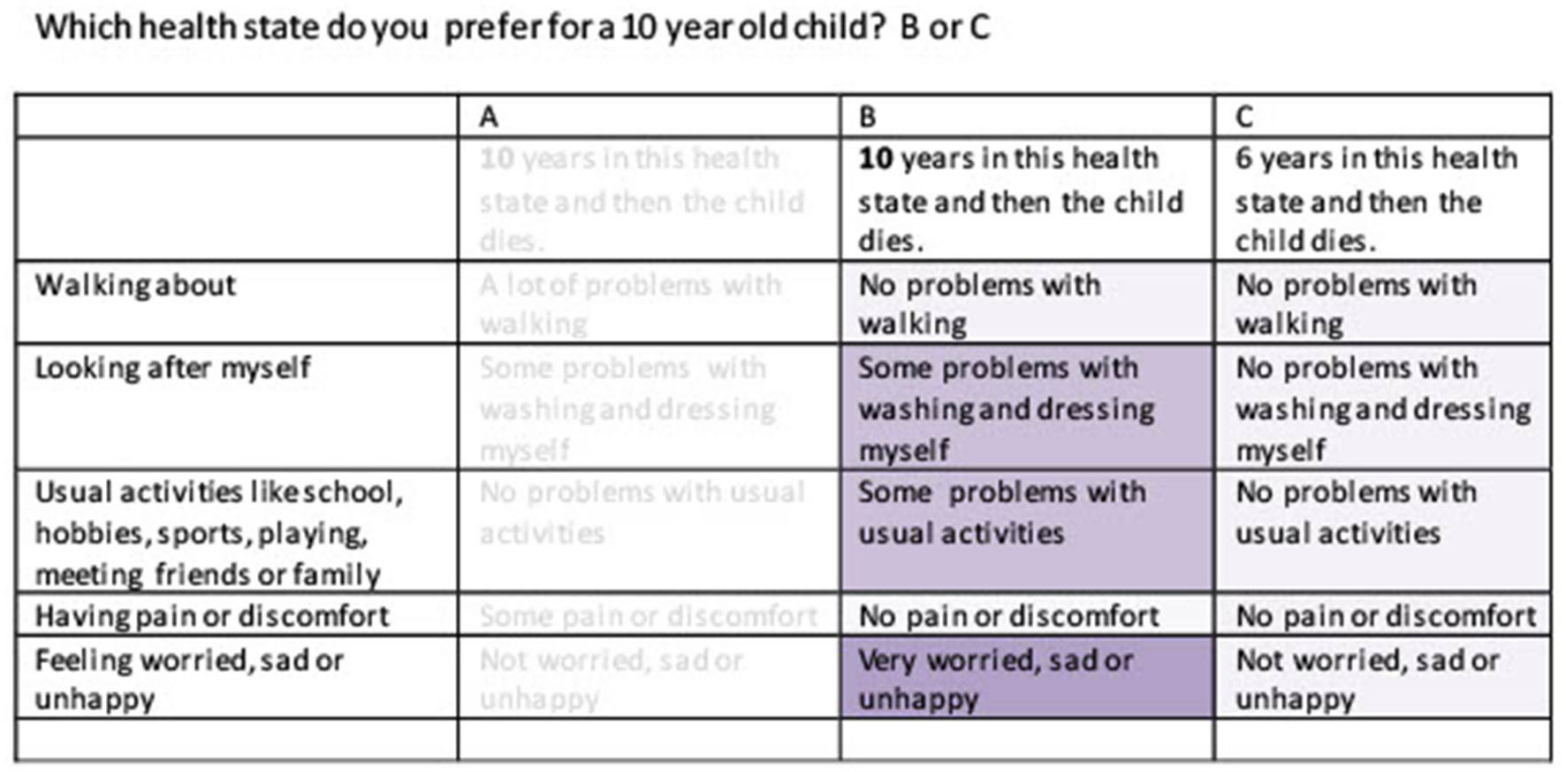
Example choice set health state B and C.

The online survey was developed by Survey Engine in both countries, with the Dutch version being translated into a Chinese version. It started with three questions regarding birth date, gender, and education. Subsequently, respondents were asked to describe their own health based on the EQ-5D-3L and the VAS scale. Then, the objective of the study was explained, and respondents were asked to fill in 15 choice tasks from a discrete choice experiment (DCE). The choice tasks were formatted as matched pairwise choices, following Jonker et al. (2017). This means that they first were asked which of two EQ-5D-Y states, A or B, they preferred for either a 10-year-old child, a 15-year-old adolescent, a 40-year-old adult, or a 70 year old (Chinese version). Both options differed in health but shared an equal life span. Next, they were asked to choose between health states B and C. C represented perfect health, i.e., no problems in any of the five EQ-5D-Y dimensions, but always offered fewer life years compared to B. To make the choice task easier, color coding was applied, with more severe problems darker colored and less severe problems lighter colored (Jonker et al., 2018a). After finishing the DCE, feasibility questions were presented, which means that respondents were asked whether they experienced any difficulties when choosing between A or B and B or C. Examples of both choice sets are presented in Figures 1, 2.

**Figure 3:**
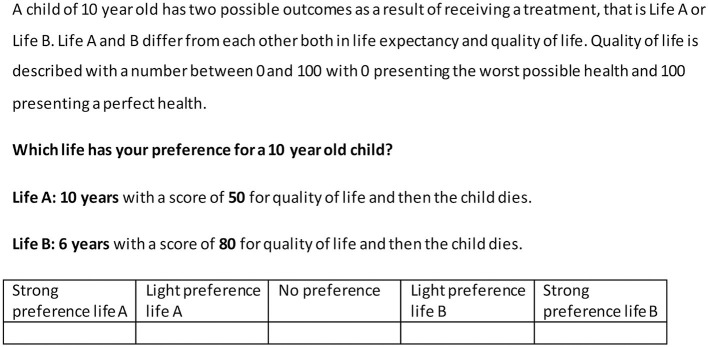
Example QALY composition task.

Next, we presented a slightly different choice task, that we dubbed a “QALY composition task”. Eight QALY composition tasks were administered. We developed the task to let responses directly tell if people weigh life years and quality of life differently for children, adolescents, adults, or older adults. The QALY composition task involved choices between different ways of achieving a similar QALY total [e.g., life A 2 years in full health (100% QoL) vs. life B 4 years in 50% QoL]. Respondents could indicate their preference for life A or life B on a 5-point Likert scale, varying from a very strong preference for life A to a very strong preference for life B. An example of a QALY task is presented in Figure 3. Eight QALY composition tasks were administered. We developed the task to explore if the relative weights attached to time and HRQoL vary for children, adolescents, adults, or older adults.

At the end of the survey, a number of background questions were asked: employment status, experience in working with children, having children, experience with serious illness of a child, experience with own health during youth, having brother(s) and/or sister(s), experience with serious illness in sibling(s), whether it would have been worse or not if the respondent would have experienced the health states described in the survey instead of the hypothetical 10, 15, 40, or 70-year-olds, what kind of child, adolescent, adult, or older adult they were thinking of when answering the choice tasks, and what kind of religion they belonged to.

### Experimental design DCE

An experimental design with 150 matched pair-wise choice questions was generated using a two-step approach. The EQ-5D-Y states featured as options A and B were selected first, subsequently, option C was added, and in a separate step, the duration levels associated with options A, B, and C were selected. This two-step approach was used to promote consistency with a UK study that used a DCE without duration (Mott et al., 2021 plenary meeting of EuroQoL). Briefly, A and B were selected using an algorithm to create a Bayesian efficient design programmed in Stata. The candidate set was restricted to pairs that had overlapping severity levels in two dimensions. The design accounted for the main effects and two-way interactions. The initial design was created without priors, but data collection was paused two times to allow interim analysis of the data. The obtained coefficients were used as priors to update a design for the next round of data collection. As mentioned above, the C alternative always referred to full health, and hence dominated A and B in terms of quality of life, but was paired with a shorter duration, implying a time trade-off question. The selection of the levels of duration associated with A and B (the same level) and with C (a shorter duration) was also informed by a Bayesian efficient design algorithm (cf.), but this part was programmed in C++ because the utility function accounted for possible non-linearities in preferences for time (i.e., discounting), which standard software packages such as NGENE or STATA could both not handle (Jonker et al., 2018b).

Blocking was applied to divide the 150 matched pairwise choice tasks into 10 blocks, with each block containing 15 pairwise comparisons.

#### Experimental design QALY composition task

The QALY composition task was constructed on the basis of an orthogonal array. The four variables linked to the orthogonal array were:

1. Life years of a (levels: 2, 4, 6, or 8)
2. Quality of life of a (levels 0.2, 0.4, 0.6, and 0.8)
3. Quality of life of b (levels 0.2, 0.5, and 0.7, 1) and
4. The ratio of total QALYs in a/b (levels 0.8, 1.0, and 1.2).

Together, these four variables were used to define the life years of B, as indicated in Table 1. The scenarios presented to respondents in the QALY composition tasks were defined by the variables in the shaded columns. Three variables were directly obtained from the orthogonal array, and the fourth (life years in option B) was computed by matching the information of the first three variables with the QALY multiplier. This procedure ensured that decision rules based on longest life, highest quality of life, or maximum number of QALYs would produce different results.

**Table 1:** Design QALY composition tasks.

**Scenarios**	**Life years A**	**Qol A**	**Qol B**	**QALY multiplier**	Life years B^*^
1	2	0.2	0.25	0.8	1.5
2	4	0.6	1	1	2.5
3	6	0.8	0.5	1	10.0
4	8	0.4	0.7	1.2	5.5
5	4	0.4	0.5	0.8	2.5
6	2	0.8	0.7	1	2.3
7	8	0.6	0.2	1	24.0
8	6	0.2	1	1.2	1.5
9	6	0.6	0.7	0.8	4.0
10	8	0.2	0.5	1	3.0
11	2	0.4	1	1	0.8
12	4	0.8	0.2	1.2	19.0
13	8	0.8	1	0.8	5.0
14	6	0.4	0.2	1	12.0
15	4	0,2	0.7	1	1.2
16	2	0,6	0.5	1.2	3.0

^*^Numbers are rounded up for convenience.

### Framing of the survey for the age groups

Exactly, the same DCE design and design of the QALY composition task were used in all arms. The only difference between arms was that respondents were asked to imagine that the health states applied for a different hypothetical person, aged 10, 15, 40, or 70 years. We used the wording of the EQ-5D-Y questionnaire to describe the health states of all arms. Only the examples mentioned between brackets for the dimension usual activities were taken from the adult version of the EQ-5D-3L for the 40-year and 70-year-old arm. For every respondent, randomization was applied per arm, per block, per choice task, and in the left-right order of the health states A and B.

### Data analysis

#### Data quality management

We retained respondents in the sample if they had completed the DCE survey and were not classified as speeders. Speeders were removed from the sample using a speeding threshold set at 530 s for the entire survey. We set this relatively low threshold to account for the fact that choice questions in a DCE repeat much of their content and to avoid undue exclusion of valid responses.

#### Discrete choice experiment

Logistic regression was used to analyze the respondent's DCE choices (STATA version 14). The parameters of the conditional logit model were estimated using maximum likelihood estimation. Conceptually, the utility that the respondent *n* obtains from alternative *j* in a choice task *t* is computed as the utility obtained from the health state characteristics X_njt_ with their accompanying preference parameters (β_n_), multiplied by the net present value (NPV_njt_) of the number of years T_njt_ associated with that health states, i.e.,


(1) $$\begin{array}{l} {\text{U}}_{\text{njt}}={\left({\text{β}}_{\text{n}.}{\text{X}}_{\text{njt}}\right)}_{.}\text{NP}{\text{V}}_{\text{njt}}+{\text{ε}}_{\text{njt}} \end{array}$$

An exponential discount function was used to compute NPV (Jonker et al., 2018b), which defines NPV by the discount rate r, i.e.,


(2) $$\begin{array}{l} \text{NP}{\text{V}}_{\text{ita}}=\left(1-\text{exp}\left(-\text{r }{\text{T}}_{\text{ita}}\right)\right)/\left(\text{exp}\left(\text{r}\right)-1\right)\;\text{if r}\neq 0 \end{array}$$

Dummy coding was applied for the levels of the EQ-5D-Y with no problems as a reference level. The coefficients from formula 1 that are associated with the dimension severity levels can be converted to the preferred scale for QALY computation, by dividing the relevant β_n_ by the preference parameter associated with years, based on the Net present value computation.

#### Feasibility

Feasibility questions for the DCE were analyzed with descriptive statistics in SPSS version 16.

#### QALY composition

The QALY composition task provided ordinal responses on a 5-point Likert scale. By arm, we computed and compared the percentages of responses in each category. We graphically display the results using horizontally stacked bars. Because minimal differences were found, no attempt was made to study differences across arms using non-parametric tests.

## Results

### Characteristics of the sample

In total, 5,126 Dutch and 4,128 Chinese respondents started the survey, with 1,730 or 2,494 respondents completing it, resulting in a response rate of 34 and 60%, respectively. A total of 496 people were excluded from the Dutch sample as speeders and 676 from the Chinese sample. After these exclusions, the Dutch sample had *N* = 438 respondents in arm 1 (10 years old), *N* = 450 in arm 2 (15 years old), and 346 (40 years old) in arm 3. The final Chinese sample had 454, 455, 454, and 455 respondents in arms 1 (10 years old), 2 (15 years old), 3 (40 years old), and 4 (70 years old), respectively. Sample characteristics are presented in Tables 2A, B. The samples were representative of the populations in terms of sex and age, although the percentage of respondents with lower education in the Netherlands was smaller compared to the population as registered by the Dutch National Bureau Of Statistics (CBS), while in the Chinese sample, the percentage of respondents with college and higher education was much higher compared to Chinese norms (CotSNPCNE, n.d.).

**Table 2:** Characteristics study samples.

	**10 year old**	**15 year old**	**40 year old**	**Dutch population**	
**A. Netherlands**					
**Age**	*N* = 438	*N* = 450	*N* = 346		
18–25	36 (8%)	38 (8%)	28 (8%)	15%	
25–35	49 (11%)	54 (12%)	39 (11%)	15%	
35–45	60 (14%)	55 (12%)	45 (13%)	15%	
45–55	84 (19%)	87 (19%)	63 (18%)	18%	
55–65	80 (18%)	82 (18%)	70 (20%)	16%	
65–75	73 (17%)	77 (17%)	57 (16%)	13%	
>75	56 (13%)	57 (13%)	44 (13%)	9%	
**Education**					
None	1 (0%)	0 (0%)	2 (1%)		
Lower	95 (22%)	107 (24%)	70 (20%)	31%	
Middle	186 (42%)	185 (41%)	165 (48%)	40%	
High	137 (31%)	139 (31%)	98 (28%)	28%	
Other	19 (4%)	19 (4%)	11 (3%)	1%	
**Sex**					
Male	192 (44%)	198 (44%)	153 (44%)	51%	
Female	246 (56%)	252 (56%)	193 (56%)	49%	
**Having children**					
Yes	272 (63%)	278 (63%)	218 (64%)		
No	163 (37%)	164 (7%)	124 (6%)		
	**10 year old**	**15 year old**	**40 year old**	**70 year old**	**Chinese norms**
**B. China**					
**Age**	*N* = 454	*N* = 455	*N* = 454	*N* = 455	
18–25	83 (18%)	78 (17%)	81 (18%)	81 (18%)	10%
25–35	110 (24%)	111 (24%)	107 (24%)	97 (21%)	17%
35–45	104 (23%)	102 (22%)	106 (23%)	97 (21%)	15%
45–55	64 (14%)	69 (15%)	67 (15%)	79 (17%)	18%
55–65	57 (13%)	55 (12%)	56 (12%)	52 (11%)	11%
65–75	30 (7%)	34 (7%)	35 (8%)	46 (10%)	7%
>75	6 (1%)	6 (1%)	2 (0%)	3 (1%)	4%
**Education**					
None	2 (0%)	1 (0%)	1 (0%)	1 (0%)	11%
Primary school	3 (1%)	5 (1%)	4 (1%)	4 (1%)	25%
Middle school	15 (3%)	17 (4%)	16 (4%)	20 (4%)	35%
High school	71 (16%)	88 (19%)	80 (18%)	93 (20%)	15%
College and above	363 (80%)	344 (76%)	353 (78%)	337 (74%)	15%
**Sex**					
Male	225 (50%)	222 (49%)	219 (48%)	239 (53%)	51%
Female	229 (50%)	233 (51%)	235 (52%)	216 (47%)	49%
**Having children**					
Yes	298 (66%)	281 (62%)	285 (63%)	286 (63%)	
No	79 (17%)	83 (18%)	70 (15%)	93 (20%)	
Unknown/missing	77 (17%)	91 (20%)	99 (22%)	76 (17%)	

### Feasibility

Tables 3A, B shows the answers related to the feasibility questions. In the Netherlands, 53% of the 10-year-old arm felt it was difficult to choose between health states A and B, compared to 45% of the adolescent arm and 34% of the adult arm. In addition, when making a choice between an impaired health state B and perfect health state C but with a shorter life duration, 58% of the respondents in the child arm and 49% in the adolescent arm answered that it was difficult to very difficult compared 43% in the adult arm. On the contrary, respondents across the four arms in China felt the degree of difficulty was similar.

**Table 3:** Feasibility questions.

	**10-year old**	**15 year old**	**40 year old**	
**A. Netherlands**				
**Difficulty choosing between health state A and B**	*N* = 438	*N* = 448	*N* = 346	
Very difficult	55 (13%)	25 (6%)	15 (4%)	
Difficult	176 (40%)	175 (39%)	103 (30%)	
Neutral	134 (31%)	171 (38%)	164 (47%)	
Easy	71 (16%)	67 (15%)	57 (16%)	
Very easy	2 (0%)	10 (2%)	7 (2%)	
**Difficulty choosing between health state B and C**	*N* = 438	*N* = 448	*N* = 346	
Very difficult	82 (19%)	66 (15%)	40 (12%)	
Difficult	172 (39%)	155 (35%)	110 (32%)	
Neutral	116 (26%)	126 (28%)	93 (27%)	
Easy	56 (13%)	79 (18%)	85 (24%)	
Very easy	12 (3%)	22 (5%)	18 (5%)	
**Would your choices have been different if the health problems concerned yourself instead of a hypothetical person?**	*N* = 435	*N* = 441	*N* = 341	
Yes, health problems for myself worse	16 (4%)	14 (3%)	23 (7%)	
Yes, loss life years for myself worse	16 (4%)	20 (5%)	25 (7%)	
Yes, health problems for myself less bad	59 (14%)	47 (11%)	10 (3%)	
Yes, loss life years for myself less bad	61 (14%)	57 (13%)	14 (4%)	
No, health problems for myself equally bad	104 (24%)	143 (32%)	163 (48%)	
No, loss life years for myself equally bad	53 (12%)	55 (12%)	49 (14%)	
I do not know	126 (29%)	105 (24%)	57 (17%)	
**B. China**				
**Difficulty choosing between health state A and B**	*N* = 390	*N* =3 76	*N* = 368	*N* = 389
Very difficult	21 (5%)	22 (6%)	11 (6%)	19 (5%)
Difficult	94 (24%)	87 (23%)	88 (23%)	86 (22%)
Neutral	126 (32%)	113 (30%)	114 (30%)	119 (31%)
Easy	113 (29%)	122 (32%)	118 (32%)	127 (33%)
Very easy	36 (9%)	32 (9%)	37 (9%)	38 (10%)
**Difficulty choosing between health state B and C**	*N* = 390	*N* = 376	*N* = 368	*N* = 389
Very difficult	23 (6%)	28 (7%)	27 (7%)	27 (7%)
Difficult	97 (25%)	81 (22%)	66 (22%)	86 (22%)
Neutral	104 (27%)	99 (26%)	101 (26%)	85 (22%)
Easy	126 (32%)	121 (32%)	140 (32%)	138 (35%)
Very easy	40 (10%)	47 (13%)	34 (13%)	53 (14%)
**Would your choices have been different if the health problems concerned yourself instead of a hypothetical person?**	*N* = 373	*N* = 361	*N* = 355	*N* = 374
Yes, health problems for myself worse	88 (24%)	96 (27%)	27% (104)	98 (26%)
Yes, loss life years for myself worse	111 (30%)	95 (26%)	26% (101)	119 (32%)
Yes, health problems for myself less worse	39 (10%)	34 (9%)	9% (44)	39 (10%)
Yes, loss life years for myself less worse	32 (9%)	31 (9%)	9% (27)	40 (11%)
No, health problems for myself equally worse	60 (16%)	56 (16%)	16% (43)	28 (7%)
No, loss life years for myself equally worse	22 (6%)	28 (8%)	8% (17)	16 (4%)
I do not know	21 (6%)	21 (6%)	6% (19)	34 (9%)

The percentage of respondents answering that their choices would not have been different if they themselves had experienced the health states rather than a hypothetical child, adolescent, adult, or older person, varied across arms in the Netherlands (Table 3A). A total of 62% of the respondents in the adult arm indicated that answering the questions for themselves would have resulted in the same responses, vs. 36% in the child arm and 44% in the adolescent arm. In the child and adolescent arms, 28 and 24% of the people considered health problems or loss of life years *less bad for themselves*, whereas, in the adult arm, respondents more often considered these issues *worse for themselves*. In China, fewer people stated that their responses would have been the same if they were asked about preferences for themselves (11–24% varying across arms), and the majority (varying between 51 and 58%) of the people in all arms state that they would consider health problems or loss of life years worse for themselves (Table 3B).

### Results discrete choice experiment

Tables 4A, B shows the results of the regression model on a latent scale for the Netherlands and China. The parameter “years” reflects the additional utility gained from a life year without health problems, before discounting, and is positive—as expected. In both countries, results show that additional life years generate utility. The interaction terms in the Dutch regression model all have the expected negative sign, except mobility level 2, showing that a deviation from full health with no problems is considered negative. The interaction terms for level 2 problems on the dimensions of self-care, usual activities, and pain/discomfort showed unexpected positive signs in China.

**Table 4A:** Results non-linear preferences on a latent scale Dutch population.

	**10 year old**		**15 year old**		**40 year old**	
	**Coefficient**	**95% CI**	**Coefficient**	**95% CI**	**Coefficient**	**95% CI**
Years	1.04	0.89; 1.19	1.12	0.98; 1.25	1.05	0.90; 1.20
Mo2^*^years	0.05	0.02; 0.08	0.06	0.04; 0.09	0.07	0.04; 0.11
Mo3^*^years	−0.07	−0.10; −0.05	−0.06	−0.09; −0.04	−0.09	−0.12; −0.06
Sc2^*^years^*^	−0.01	−0.04; 0.01	0.00	−0.02; 0.03	−0.01	−0.04; 0.02
Sc3^*^years	−0.12	−0.15; −0.09	−0.13	−0.15; −0.10	−0.14	−0.17; −0.11
Ua2^*^years	−0.06	−0.09; −0.04	−0.07	−0.10; −0.05	−0.05	−0.08; −0.03
Ua3^*^years	−0.32	−0.37; −0.28	−0.32	−0.36; −0.28	−0.28	−0.32; −0.24
Pd2^*^years	−0.14	−0.18; −0.11	−0.10	−0.13; −0.08	−0.08	−0.11; −0.06
Pd3^*^years	−0.54	−0.61; −0.47	−0.49	−0.54; −0.43	−0.44	−0.50; −0.38
Ad2^*^years	−0.19	−0.22; −0.15	−0.17	−0.20; −0.14	−0.16	−0.19; −0.12
Ad3^*^years	−0.64	−0.72; −0.56	−0.63	−0.69; −0.56	−0.57	−0.64; −0.49
Discount rate	0.25	0.22; 0.28	0.23	0.20; 0.25	0.22	0.19; 0.25

^*^Indicate that this is an interaction between the domain like mobility and years as described under the heading results discrete choice experiment.

**Table 4B:** Results non-linear preferences on a latent scale Chinese population.

	**10 year old**		**15 year old**		**40 year old**		**70 year old**	
	**Coefficient**	**95% CI**	**Coefficient**	**95% CI**	**Coefficient**	**95% CI**	**Coefficient**	**95% CI**
Years	0.27	0.23; 0.31	0.29	0.25; 0.33	0.33	0.29; 0.37	0.23	0.19; 0.27
Mo2^*^years	−0.01	−0.03; 0.02	−0.01	−0.03; 0.02	0.01	−0.02; 0.03	−0.01	−0.04; 0.02
Mo3^*^years	−0.08	−0.10; 0.05	−0.10	−0.14; 0.06	−0.11	−0.15; −0.07	−0.09	−0.13; −0.05
Sc2^*^years	0.04	0.01; 0.08	0.03	0.02; 0.08	0.06	0.03; 0.09	0.02	−0.01; 0.05
Sc3^*^years	−0.02	−0.04; 0.03	−0.02	−0.05; 0.01	−0.05	−0.08; −0.02	−0.06	−0.10; −0.02
Ua2^*^years	0.01	−0.02; 0.03	0.01	−0.02; 0.05	0.03	0.00; 0.06	0.03	−0.00; 0.06
Ua3^*^years	−0.04	−0.08; 0.00	−0.05	−0.08; 0.00	−0.05	−0.09; −0.01	−0.05	−0.09; −0.02
Pd2^*^years	0.01	−0.02; 0.03	0.01	−0.02; −0.03	0.00	−0.02; 0.03	0.02	−0.01; 0.05
Pd3^*^years	−0.08	−0.13; 0.00	−0.10	−0.14; 0.04	−0.10	−0.14; −0.06	−0.10	−0.12; −0.06
Ad2^*^years	−0.00	−0.03; 0.02	−0.03	−0.06; 0.00	−0.00	−0.03; 0.02	−0.03	−0.04; 0.00
Ad3^*^years	−0.14	−0.17; −0.11	−0.13	−0.17; −0.09	−0.13	−0.18; −0.08	−0.13	−0.17; −0.10
Discount rate	0.29	0.25; 0.33	0.33	0.28; 0.38	0.32	0.28; 0.36	0.32	0.27; 0.37

The estimated discount rate *r* varied between 0.22 and 0.25 across the arms in the Netherlands and was ~0.30 in China in all four arms, suggesting strong discounting of future health outcomes.

**Figure 4:**
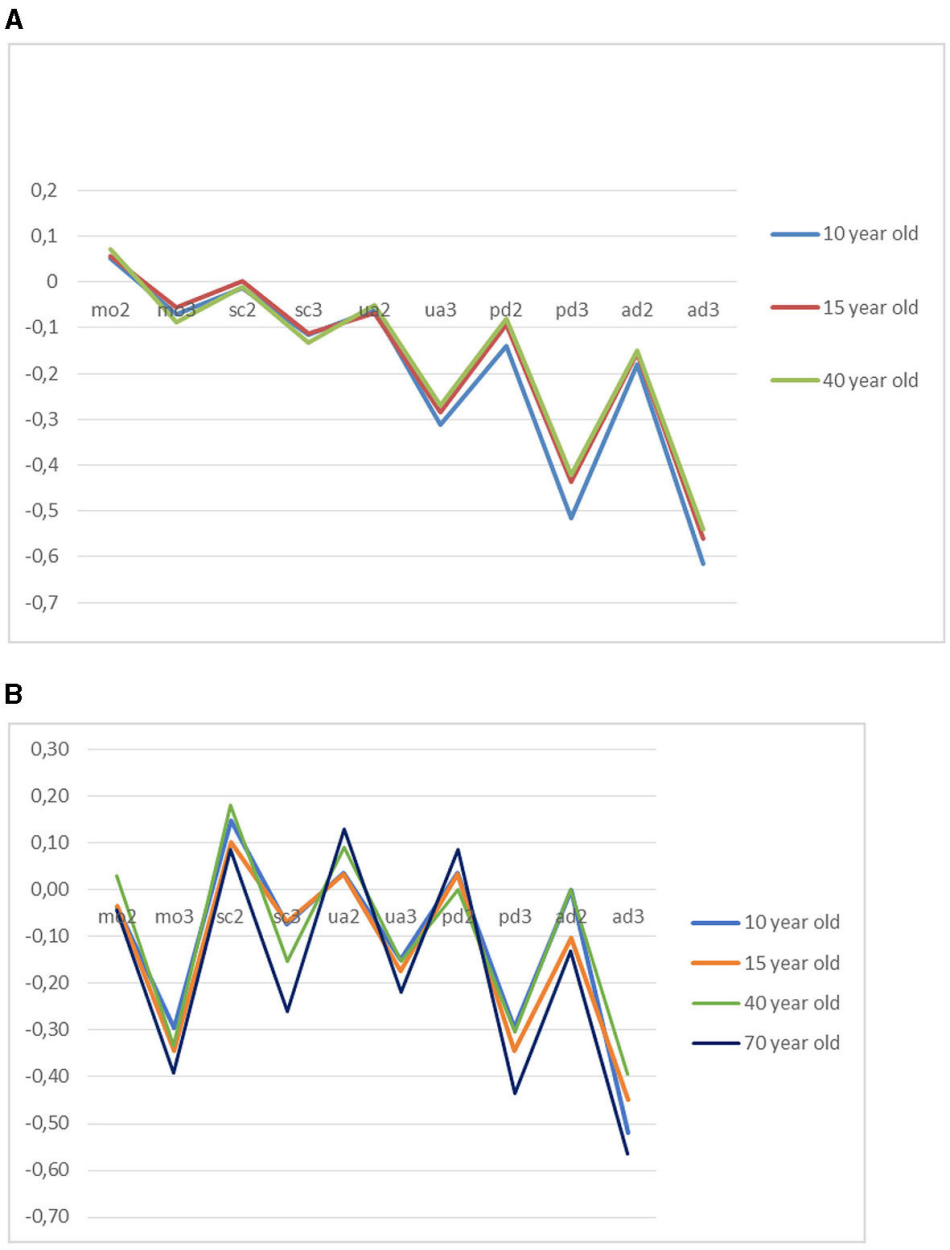
**(A)** Utility decrements per EQ-5D-Y dimension severity level in the Netherlands. **(B)** Utility decrements per EQ-5D-Y dimension severity level in China.

Figures 4A, B presents the results on a QALY scale (coefficient interaction term divided by coefficient years). Across arms in the Netherlands, we found a high level of agreement on the health state values, except for the dimensions of pain and discomfort and anxiety/depression; respondents traded-off *more* time to avoid these problems for children than for adults. The Chinese results showed that respondents traded-off more time to avoid severe problems in the 70-year arm.

The difference in values for the worst health state (33,333) resulted in −0.630 for children, −0.452 for adolescents, and −0.452 for adults in the Netherlands. On the contrary, older adults in China have a value of −0.870 for the worst state, followed by adolescents (−0.370), children (−0.340), and adults (−0.320).

### QALY composition

**Figure 5:**
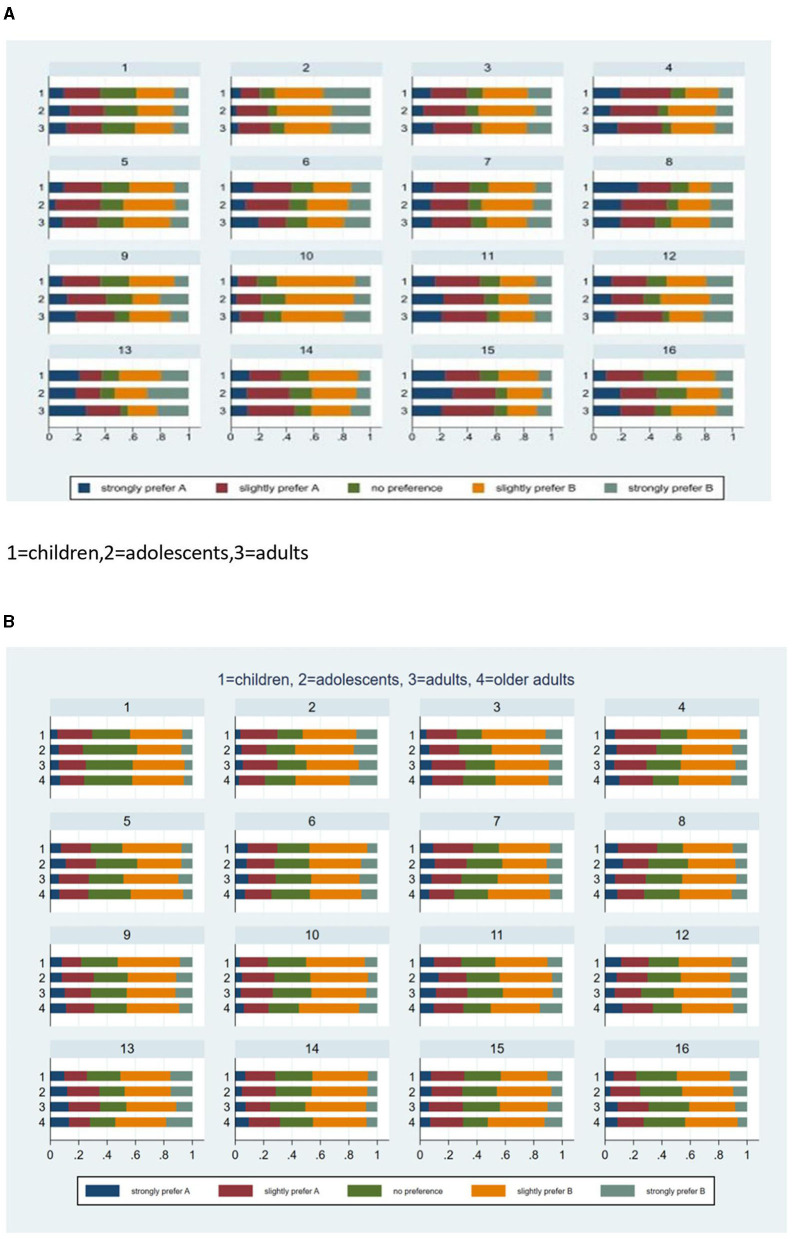
**(A)** Distribution of likert responses by scenario in the Netherlands. **(B)** Distribution of likert responses by scenario in China.

Figures 5A, B presents the distribution of the Likert responses by QALY composition task. We found no clear pattern of differences across arms in both countries. The distribution over response options varied most when the life years or quality of life were at the extremes of the spectrum. In the Netherlands, the only distinction between arms was that the percentage of responses in the third response category, indicating no preference for A or B, seemed to be the largest when the questions concerned a 10-year-old child. The Chinese results showed a larger percentage of respondents, indicating no preference between life A and life B compared to the Dutch data, with similar or even less clustering in the child's arm on the no preference option.

## Discussion

This study examined the impact of framing of age on values for EQ-5D-Y health states in the Netherlands and China. We tested this issue using a DCE duration approach and a task that assessed preferences for QALY composition. The empirical findings indicated that the values derived from the DCE duration task were similar in the Netherlands for children, adolescents, and adults (except for “pain”) and varied more for children, adolescents, adults, and older adults in China, where the lowest values were found in the group stating preferences for a 70-year-old person. Dutch people comparatively traded-off more time to avoid pain in children than for adults, resulting in lower values, while Chinese people were more willing to trade-off time to avoid any type of severe problem in the elderly compared to the other arms. The QALY composition task showed no clear differences in values for health across age groups.

No evidence for age dependency of health state values was found in the Netherlands. Our results for the 10-year-old arm are consistent with Kreimeier's TTO results (Kreimeier et al., 2018). Based on international results, Kreimeier reported that TTO values applied to children generally were higher compared to values of adults, but in that study, the Dutch results were an exception. In the Netherlands, people gave a lower TTO value to a health state when it concerned a 10 years old compared to themselves (Kreimeier et al., 2018). This indicates that Dutch respondents are prepared to trade-off life years against the quality of life for children. In our research, the results also showed that respondents were prepared to trade-off more time to avoid pain in children than in adults, resulting in lower values, although generally, the agreement of health state values for different ages was quite strong. While the congruence between studies supports the validity of our findings, care should still be taken when generalizing our results to other countries. Stronger evidence for age dependency of values was found in China, where the inclusion of the 70-year arm increased the contrast between groups.

Our estimation of health state utilities followed a state-of-the-art DCE duration approach, requiring a multiplicative utility function that involves a non-linear discount function. The estimated discount rates indicated that respondents valued quality of life in the short term more compared to the long term, which was anticipated, and as argued by Jonker and Bliemer (2019), valid health state utility values can only be obtained if the model adequately accounts for such time preferences. The estimated discount rates were, however, relatively high when compared to the standard rates usually applied in economic evaluations, especially in China. While the discount rates were still within the range of previously estimated discount rates for health-related outcomes (Attema et al., 2018), their reliability needs to be established in the future research. A limitation of the DCE duration method is that the best way to account for time preferences, especially in the presence of discounting, has not been identified. Discount rates can be computed in different ways. Models that account for non-linear time preferences are complex and have not been implemented yet in the standard software that we used for choice modeling, and this limits the modeling options (e.g., we cannot simultaneously account for preference heterogeneity and for non-linear time preferences). Furthermore, this way of assessing preferences places high demands on the design, necessitating interim design updates to ensure that the design is based on adequate priors, and the end results may still depend on the data quality obtained along the way. We excluded speeders *post-hoc*, not before design updates.

If we are examining preferences for a subject like a trade-off between life years and quality of life, we also need to carefully consider what advantages and disadvantages different valuation methods may have when used in such a context. We consider it possible that the use of TTO poses even greater challenges than the DCE of the required accuracy in rating health states and direct assessment. A specific result that may be worth noting is the larger clustering of responses in the child arm vs. the other arms on the no preference answer option in the QALY composition task in the Netherlands. This might indicate that a larger fraction of respondents in the child arm feel uncertain when trading-off quality of life and life years. However, it is also possible that respondents are neutral about their preference for either one of the options and consider them equivalent. Either way, it shows that more respondents in the child's arm were reserved when making a choice. However, it appears that the Chinese results showed a reversed pattern, with more respondents in the child's arms who were more certain to make a decision. The possible explanation may be a cultural difference: paternalism is more prevalent in China.

The findings of this study may be taken into consideration for future updates of the EQ-5D-Y valuation protocol. EQ-5D-Y values are currently elicited from adults who value health states accruing to a 10-year-old child (Ramos-Goñi et al., 2020). This study reflects on the appropriateness of using a specified age (here, 10 years of age) in the elicitation of values that are used across a wider age group by varying the specified age. Age dependency of values was limited in the Netherlands, suggesting that values elicited for a 10-year-old child may also be validly applied for a 15-year-old. However, in China, the values for 70-year-olds differed strongly from the values for other ages, suggesting that the appropriateness of using a fixed, specified age may be questioned. Moreover, many respondents indicated that their choices would have been different if the health state had been experienced by themselves rather than by someone else. This finding is in line with results from other studies (Lipman et al., 2021; Reckers-Droog et al., 2022). More research on the sensitivity of values to age and perspective is warranted.

## Conclusion

Age dependency was observed in the stated preferences for hypothetical health states. The magnitude and direction of age effects in values seemed dimension- and country-specific. In the Netherlands, we found a few differences in dimension-specific weights elicited for 10- and 15-year-olds compared to 40-year-olds, but the overall age dependency of values was limited. A stronger age dependency of values was observed in China, where values for 70-year-olds differed strongly from the values for other ages. The appropriateness of using existing values beyond the age range for which they were measured needs to be evaluated in the local context.

## Data availability statement

The raw data supporting the conclusions of this article will be made available by the authors, without undue reservation.

## Ethics statement

The studies involving human participants were reviewed and approved by the Ethics Committees of the University Maastricht and Institutional Review Board of Fudan University School of Public Health. Written informed consent from the participants was not required to participate in this study in accordance with the national legislation and the institutional requirements.

## Author contributions

BE, PW, ES, MFJ, SE, MJ, and CD: conceptualization and writing and critical review. BE, PW, MFJ, and ES: design and analysis. All authors contributed to the article and approved the submitted version.
